# Needs, Preferences, and Values during Different Treatment Decisions of Patients with Differentiated Thyroid Cancer

**DOI:** 10.3390/jpm11070682

**Published:** 2021-07-20

**Authors:** Anna Koot, Romana Netea-Maier, Petronella Ottevanger, Rosella Hermens, Peep Stalmeier

**Affiliations:** 1 Radboud Institute for Health Sciences, Department for Health Evidence, Radboud University Medical Center, 6500 HB Nijmegen, The Netherlands; peep.stalmeier@radboudumc.nl; 2 Department of Internal Medicine, Division of Endocrinology, Radboud University Medical Center, 6525 GA Nijmegen, The Netherlands; romana.netea-maier@radboudumc.nl; 3 Department of Internal Medicine, Division of Oncology, Radboud University Medical Center, 6525 GA Nijmegen, The Netherlands; nelleke.ottevanger@radboudumc.nl; 4 Radboud Institute for Health Sciences, Scientific Institute for Quality of Healthcare (IQ Healthcare), Radboud University Medical Center, 6500 HB Nijmegen, The Netherlands; rosella.hermens@radboudumc.nl

**Keywords:** differentiated thyroid cancer, information needs and preferences, focus group interview

## Abstract

Background: The purpose of this study was to identify the needs, preferences, and values of patients with differentiated thyroid cancer (DTC) and the physicians treating patients with DTC regarding two different treatment decisions, namely: the extent of primary surgery (low-risk patients) and the tyrosine kinase inhibitor (TKI) treatment (high-risk patients). Methods: A qualitative study was conducted. There were two physician focus groups discussing the extent of primary surgery. One included endocrinologists (n = 4) and surgeons (n = 5), and the other included nuclear medicine physicians (n = 3) treating patients with low-risk DTC. The physicians focus group discussing waiting or starting TKIs included endocrinologists (n = 2) and oncologists (n = 5) treating patients with advanced radioactive iodide (RAI) refractory DTC. Moreover, one patient focus group per treatment decision took place. In total 13 patients and 19 physicians participated. Interviews were audio-taped, fully transcribed verbatim, and analyzed. Results: Several themes were identified. Patients, but not physicians, mentioned the importance of a strong doctor–patient relationship. Patients in both treatment decision groups wanted to receive more detailed information, whereas physicians preferred providing more general information. Patients in the TKI decision group focused on palliative care, whereas physicians focused more on the effect and benefit of TKIs. Conclusions: Considering the identified themes in DTC, based on the patients’ needs, preferences, and values, enables us to improve doctor–patient communication and to develop decision support tools.

## 1. Introduction

Most patients with low-risk differentiated thyroid cancer (DTC) have an excellent long-term prognosis [1,2,3,4]. Up to 30% of DTC patients develop recurrent disease and/or distant metastases. Patients with distant metastases have a five-years survival rate of approximately 50%, which is worse in those with advanced radioactive iodide (RAI) refractory DTC [5,6]. Therefore, considering the relatively high survival rates for both low-risk and high-risk DTC survivors, maintaining long-term quality of life (QOL) is important.

Recently, clinical practice shifted towards more individualized approaches, and patients are involved in trade-offs between the harms and benefits of different approaches [7]. For patients with low-risk DTC and patients with RAI refractory DTC, the optimal treatment is debated, as insufficient evidence is available regarding the harms and benefits of treatments in relation to the oncological outcome. As such, some low-risk DTC patients might undergo overtreatment, thus negatively affecting QOL. The American Thyroid Association (ATA) guidelines suggest considering patient preferences, as for some low-risk patients, thyroid lobectomy (TL) alone may be a sufficient initial treatment [8,9]. Similarly, for asymptomatic or mildly symptomatic RAI refractory DTC patients, premature starting of tyrosine kinase inhibitors (TKIs) may expose patients to side-effects and worsening QOL. The recent European Thyroid Association (ETA) guidelines stated that the decision to start TKIs should include patient-related medical factors and patient preferences with respect to treatment goals and values, as well as the acceptance of adverse effects [10]. However, a recommendation on how to shape the communication process is lacking.

Doctor–patient communication could be improved to consider the patients’ perspective by using shared decision making (SDM). Accordingly, physicians provide patients with information on existing options and consider patients’ needs in order to enable a personalized treatment choice [11]. In this process, important life goals are involved, and these should be explored together with the patient [12,13,14,15]. In practice, however, talking about values is difficult, and values are discussed in a minority of decision-making consultations [11]. Moreover, exploring values is complicated by different perspectives of physicians and patients [16].

To facilitate SDM in doctor–patient communication, it is important to determine the patients’ needs. Decision-support tools, such as decision aids and values clarification exercises, exist to inform patients and help them explore their values [17,18]. These tools can be applied in clinical practice. These instruments are not yet available for all patients with DTC. For developing such tools, knowledge about patients’ and physicians’ needs is required. Previous studies have explored the patients’ needs in patients with papillary microcarcinoma (PMC) [19,20,21,22,23] and in patients with larger low-risk DTC (>1 cm) requiring treatment [24,25,26,27,28,29,30]. However, particularly the differences between patients’ and physicians’ needs have not previously been investigated.

To reduce miscommunication between patients and their physicians, we aimed to identify the needs of DTC patients in two different treatment decision groups in preference-sensitive decision making, as stated in the ATA and ETA guidelines [9,10]: (1) the extent of primary surgery, including the need for subsequent ablation of thyroid remnants with RAI in patients with low-risk DTC (>1 cm), and (2) starting TKIs in patients with advanced RAI refractory DTC. Our aim was to, on the one hand, address the differences and similarities between the two treatment decision-making groups (regarding low-risk and high-risk patients), and on the other hand, between perspectives of physicians and patients for both decisions. In addition to previous studies, more attention was given to values communication, given the difficulty in exploring values and to help physicians to discriminate between their own and patients’ values.

## 2. Materials and Methods

### 2.1. Study Design

We performed a qualitative study using semi-structured focus group interviews. The aim of the interviews was to identify in-depth needs, preferences, and values of DTC patients in two different treatment decision groups and in physicians treating DTC patients. This study is part of the COMBO study (COMmunication Booster, NCT03905369), aiming to develop, evaluate, and implement decision-support tools for DTC patients. Twelve hospitals (six academic and six non-academic) in the Netherlands participated. The Dutch patient association “Schildklier Organisatie Nederland (SON)” was also involved. The Medical Ethical Committee (CMO) of the region Arnhem–Nijmegen approved the study protocol (MEC-2018-4521). The study is in agreement with the COREQ checklist [31].

### 2.2. Setting

In the Netherlands, DTC patients undergo treatment in both academic (high- and low-risk patients) and non-academic hospitals (mainly low-risk patients) involving multidisciplinary teams of specialists. Long-term follow-up is generally carried out by endocrinologists. Patients with advanced RAI refractory disease requiring TKI treatment are followed-up by oncologists.

### 2.3. Participants

#### 2.3.1. Patients

Two treatment decision-making groups were involved, namely: (1) patients with low-risk DTC according to the ATA criteria [9] who had surgery, and (2) patients with advanced RAI refractory DTC who started or considered TKIs. The inclusion criteria for the low-risk group were being diagnosed with DTC, having been treated with surgery within one year, and being capable of understanding their treatment trajectory as judged by their physician. Inclusion criteria for the advanced disease group were patients who started or considered TKIs within one year and were capable of understanding their treatment trajectory as judged by their physician. Six academic hospitals with expertise in DTC treatment throughout the Netherlands, as well as the Dutch patient association SON, selected patients for participation in the interviews. After physicians asked their patients to participate in the interviews, participants were approached by the researcher (A.K.) by telephone. Participation was voluntary. All participants provided written informed consent alongside answers to some demographic questions.

In total, two patient focus groups were organized. One focus group included low-risk DTC patients (n = 6) discussing thyroid lobectomy or total thyroidectomy, including the need for the subsequent ablation of thyroid remnants with RAI. The other focus group included patients with advanced disease (n = 7), discussing the watchful waiting approach or starting with TKIs decision. The patient focus group interviews took place in the main investigating center (Radboud University Medical Center). The interviewee had no treatment relationship with the participants.

#### 2.3.2. Physicians

Physicians (endocrinologists, surgeons, nuclear medicine physicians, and oncologists) from the above-mentioned expertise centers with extensive expertise on the treatment of patients with DTC were approached by the researcher (A.K.) by email. All physicians provided verbal informed consent and answered several questions regarding their clinical experience. In total, three physician focus groups were organized. To discuss the thyroid lobectomy or total thyroidectomy decision, two focus groups were organized, as follows: one included endocrinologists (n = 4) and surgeons (n = 5), and one included nuclear medicine physicians (n = 3) treating patients with low-risk DTC. To discuss the decision between watchful waiting or starting with TKIs, another focus group was held, including endocrinologists (n = 2) and oncologists (n = 5) treating patients with advanced RAI refractory DTC.

### 2.4. Data Collection

An expert panel developed an interview guide (Table A1). For both patients and physicians, the interview guide contained three sections, open-ended questions, and optional questions to elaborate each topic. The three sections referred to the diagnostic, treatment, and evaluation phases of DTC care. The interviewer (A.K., first author, female, MD endocrinology, PhD student, trained in interviewing techniques by a qualitative research expert) started by explaining the process of the interview. Next, open-ended questions, not provided in advance, were asked concerning the information given by the physician during the consultation in the specific treatment phase. The open-ended questions, focused on needs, preferences, and values; communication with the health care provider; strong and weak points of the received health care; and points to improve the current health care. Finally, patients were invited to give their opinion about the content to be included in a decision-support tool. The focus group interviews were conducted between May and December 2019. All of the focus group interviews lasted between 26 and 94 min and were audio-taped. One additional observer attended each focus group and field notes were made during the focus group interviews (R.N.-M. or P.O.). A pilot for the interview was performed with the first and fourth author (female, senior researcher, experience with qualitative research). Patients did not receive questions in advance and were not informed about the use of the framework to analyse the data using the Picker domains [32,33].

### 2.5. Analysis

First, all five focus group interviews were transcribed verbatim and qualitatively analyzed using ATLAS.ti, 8.4.15 [34]. Two researchers (A.K. and a second coder; female, experienced coder) independently analyzed all of the transcripts. The perspectives of patients and physicians and the two different treatment decisions were analyzed separately. The eight-dimension Picker domains were used as a basis for our analyses (Table A2) [32]. Expressed needs, preferences, and values were categorized into one of the eight Picker domains, particularly involvement in decisions and respect for preferences, coordination and integration of care, clear information, and communication and emotional support. All interviews were open coded independently by both researchers. Open coding allows for an exploration of the ideas and meaning that were contained in the raw data. Once codes were created using open coding, they were analyzed using the axial coding process. This analysis enabled researchers to identify connections between the codes [35]. For axial coding, two concept coding trees were made, one for the surgery decision and one for the TKI decision. Both researchers could add, remove, or move the codes of the coding tree. The codes were compared and discussed until a consensus was reached. Thereafter, the codes were categorized into similar themes and subthemes within one of the domains. We aimed to fit all themes into the Picker domains, and a new domain was proposed if the codes would not fit.

## 3. Results

**Figure 1:**
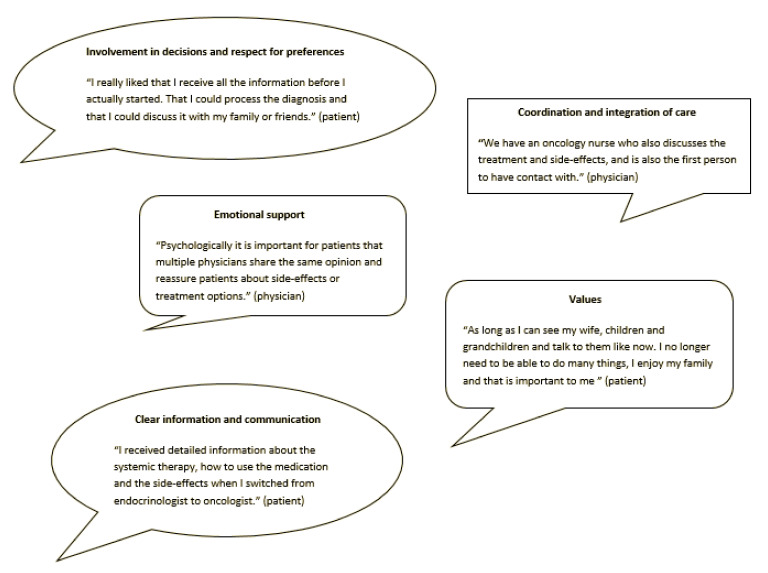
Quotes expressed by patients with DTC and by physicians treating patients with DTC for each Picker domain.

All of the invited patients and physicians agreed to participate. The patient and physician characteristics are displayed in Table 1. Sixteen themes and sixty-three subthemes were categorized for low-risk DTC patients and their respective physicians. Fifteen themes and thirty-eight subthemes were categorized for patients with advanced DTC and their respective physicians. The themes and subthemes fitted within four of the eight Picker domains (Table A2) [32]; one additional domain occurred, namely values (Table 2, Table 3, Table 4 and Table 5 and Figure 1). Table 2 shows the qualitative results for the surgery decision regarding “thyroid lobectomy or total thyroidectomy” and Table 3 shows several quotes fitted to the corresponding themes for the decision regarding “thyroid lobectomy or total thyroidectomy”. Table 4 shows the qualitative results for the decision “to wait or start with TKIs”, and Table 5 shows several quotes fitted to the corresponding themes for the decision “to wait or start with TKIs”. The identified domains were as follows.

### 3.1. Involvement in Decisions and Respect for Preferences

The importance of a good doctor–patient relationship was most often mentioned by patients with advanced RAI refractory DTC. Patients indicated the importance of discussing the treatment steps with family and friends, and listening to patients’ needs and preferences. They indicated needing a doctor who takes care and is available most of the time. Integrity and mutual respect were necessary for a good doctor–patient relationship. For patients treated with surgery, it was important to be involved in their treatment process and to discuss the options with their physician. The patients in both treatment decision groups needed time to cope with the diagnosis and its consequences. Physicians in both treatment decision groups mentioned that patient involvement in the treatment process was important. The difference between patients and physicians in this domain was the importance of a good doctor–patient relationship. This theme was rarely mentioned by physicians.

### 3.2. Coordination and Integration of Care

Themes mentioned by patients and physicians for both treatment decisions were the involvement of a multidisciplinary team and information process. It was important to have a contact person for questions and problems. Patients and physicians for both decisions mentioned that they considered an oncology nurse as the most suitable multidisciplinary team member. A tumor board should be available to discuss difficult cases with other healthcare providers. For both patients and physicians, paper and digital ways of informing were considered equally important, and adequate ways to inform patients. In this domain there were no differences between patients and physicians.

### 3.3. Clear Information and Communication

Patients in both decision groups needed clear, honest, and complete information. They also wanted to receive detailed information. The two important themes for patients in the surgery decision group were general information about DTC and treatment options. For patients in the TKI decision group, general information about advanced RAI refractory DTC and medication was important. In both groups, patients were not at all or only slightly satisfied with the amount of information received. They wanted to receive more information. Patients in both groups felt they were not involved in decision making, “there was nothing to choose”. Important themes for physicians discussing the surgery decision also involved general information about DTC and treatment options. Physicians mentioned most often “giving information about the prognosis”, especially if patients were considered to benefit from treatment with TKIs. The main difference in this domain was the detailed information patients wanted to receive, whereas physicians wanted to give more general and short information. In the TKI treatment decision group, physicians wanted to give information about the benefit of therapy, whereas patients were more focused on palliative care.

### 3.4. Emotional Support, Empathy and Respect

Patients mentioned the importance of offering psychological care. In both decision groups, emotional support and the involvement of family was important, as well as reassurance by health care providers. In both groups, patients felt reassured by their physician, they also felt that there was sufficient psychological support. Only physicians in the TKI decision group mentioned reassurance by health care providers. The vast majority of involved physicians did not mention emotional support or psychological care, which was the main difference between patients and physicians in this domain. Although few physicians did not mention it often, they apparently succeeded in reassuring the patients.

### 3.5. Values

Values were an important part of health care decisions, “what matters to me”. For patients who started or considered TKIs, the values mentioned most often involved functioning in daily life, for example, maintaining QOL and family milestones. In the surgery decision group, values regarding medical outcomes involved recovery and physical changes after treatment. In addition, the value “I need surgery, there is something in my body that does not belong there” was often mentioned. Physicians in the TKI decision group believed that values about daily life were important. Physicians in the surgery decision group mentioned medical values most often. The main difference was that patients wanted to discuss values more.

## 4. Discussion

This qualitative study investigated the perspectives of patients with low-risk (>1 cm) DTC and advanced tumors, regarding the extent of surgery or starting TKIs. We studied the differences between physician and patient perspectives, as well as the differences and similarities between the aspects involved in these two decisions. The main themes emerging from both patient and physician interviews were the involvement of a multidisciplinary team and a way of being informed, general information on DTC and treatment options, reassurance by health care providers, medical values, and values regarding functioning in daily life. The main differences in perspective between patients and physicians were that only patients mentioned the importance of a good doctor–patient relationship, patients desired detailed information about their diagnosis, patients focused on palliative treatment whereas physicians focused on the benefit of therapy, and only patients mentioned the need for psychological care and emotional support. These differences provide valuable information on the perspective of patients with DTC and physicians that can be used when designing decision aid instruments. These differences are elaborated below.

Regarding the doctor–patient relationship, patients in both treatment decision groups appreciated a good doctor–patient relationship, whereas physicians did not mention this often. Pitt et al. also showed that patients with low-risk DTC (>1 cm) wished for a strong patient–surgeon relationship, in line with the findings in patients with other malignancies [24]. This was particularly important for patients with DTC, who remained in follow-up and required medical guidance and support for many years, even after remission.

Regarding the desire to receive detailed information, patients from both decision groups wanted to receive more detailed information about DTC, treatment options, and aftercare, whereas physicians from both decisions groups preferred providing more general and short information. We hypothesize that this difference arises from the fact that physicians are limited with respect to the available consultation time and have concerns about how much of the information is remembered. However, ensuring that the information is individualized and patients are involved in the treatment process was a strong preference of our participants. This preference is corroborated by studies focusing on needs in DTC survivors after primary treatment. Such studies show that survivors usually receive information related to their diagnosis, prognosis, and primary treatment; however, information on long-term effects, recurrence, and aftercare is scarce [25,26,27,28,30]. Likewise, a systematic review of Hyun et al. showed that cancer survivors in general perceive many unmet needs, and these needs extend to aftercare [29]. Previous studies among other cancer types also showed the importance of providing patients with disease specific information about cure, spread of disease, and side effects. There are some possible explanations for this need for information: (1) it can increase trust in the caring physician and reduce possible feelings of uncertainty and doubt, and (2) it might be used as a coping strategy to gain control and to understand what is happening to body and mind [36,37,38,39].

Regarding the difference in the TKI decision group, physicians focused more on the effect and benefit of TKIs, whereas patients were focused more on palliative care. Physicians may focus more on the effects and benefits of TKIs because they prescribe TKIs when they believe there is a positive effect and benefit of therapy. We hypothesize that the physicians’ knowledge of the progression free survival (PFS) benefit shown in patients treated with TKIs in the randomized controlled trials plays an important role in this result. This could particularly be the case when patients may not be sufficiently aware of these results [40,41]. Another explanation for the differences in focus is that patients’ awareness of having an incurable disease might generate more thoughts regarding their quality of life and the availability of palliative care. No other studies focused specifically on the needs and preferences in patients with advanced metastatic DTC. Because DTC is generally a slowly progressive cancer type, patients with metastatic disease often have long-time survival while maintaining a good QOL. Therefore, a comparison with other patients with disseminated malignant tumors could not be used to corroborate our findings, which highlights the relevance of the present DTC-specific findings.

Our results indicate the importance of talking with patients about their values: values regarding treatment and decision, and values about daily life were mentioned by both patients and physicians. Identifying such values is relevant because, in practice, values are voiced or discussed in a minority of consultations [11], and to talk about values is the most difficult part of doctor–patient communication. Other studies have showed the importance of talking about values, e.g., the systematic review of Hyun et al. found that psychosocial information and supportive care needs may be insufficiently met in DTC survivors [29]. The emotional reaction “I need surgery, there is something in my body that does not belong there” was also found by Pitt et al. [39]. In other cancers, the importance of the involvement of family and friends was also found [36,37,38].

## 5. Strengths and Limitations

A strength of our study is that, on the one hand, it contrasts differences and similarities between two treatment decision-making groups (regarding low-risk and high-risk patients), and on the other hand, it contrasts differences between the perspectives of physicians and patients for both decisions. Our study has some limitations. Because of logistic difficulties, only one focus group interview per treatment decision could be organized. Therefore, our results may not give an understanding of all of the issues involved [42]. Furthermore, physicians and patients with DTC in other cultural or geographical settings may have needs that we did not identify, which could impact generalizability. In general, the results of a qualitative study cannot be generalized, although the results can be of major importance for the specialists in the field. This underscores the need for similar cross-cultural validation studies in other countries.

## 6. Conclusions

In conclusion, this study illustrates the needs, preferences, and values in patients with DTC in two different treatment decision groups in both low-risk and high-risk patients. While many of these are recognized and are overlapping with those of the physicians treating patients with DTC, some are clearly different and potentially not sufficiently addressed in daily practice. Communication may be improved by (1) meeting patients’ needs with respect to stage-specific information provision about the disease and its consequences, (2) raising awareness among the physicians to inquire about and address patients’ needs with respect to emotional and psychological support, and (3) addressing patients’ concerns about palliative care. This may help physicians to improve their communication and better meet patient needs. The results of this study can also be used to develop decision support tools to make current doctor–patient communication more SDM-based.

## Figures and Tables

**Table 1:** Characteristics of participating patients and physicians.

Characteristics of Patients (n = 13)	Number n(%)
Sex:	
Male	7 (54)
Female	6 (46)
Age, mean (range), y	57 (31–84)
Caucasian	13 (100)
Married/living together	13 (100)
Educational level:	
High school or less	2 (16)
Vocational education	11 (84)
University	0 (0)
Treatment:	
Surgery	6 (46)
Thyroid lobectomy	3 (50)
Total thyroidectomy	3 (50)
Average time from surgery (mean), m	10.5
Complication rate	1 (17)
TKIs	7 (54)
Not started	4 (57)
Started	3 (43)
Average time from progressive advanced disease (mean), m	6.9
Site of distant metastases	
* Lung	6 (60)
* Bone	3 (30)
* Other	1 (10)
Characteristics of physicians (n = 19)	Number n(%)
Sex:	
Male	7 (37)
Female	12 (63)
Working in university hospital	15 (100
Medical specialty:	
Endocrinologist	6 (32)
Oncologist	5 (26)
Surgeon	5 (26)
Nuclear medicine physician	3 (16)
Experience in years	
0–10	1 (5)
11–20	11 (58)
>20	7 (37)

y: years, m: months; * site of distant metastases

**Table 2:** Qualitative results for the surgery decision regarding “thyroid lobectomy or total thyroidectomy”. Expressed needs, preferences, and values of patients and of physicians treating patients with DTC.

Information Needs for Surgery Decision Making			
Domain	Theme		Description of Corresponding Items
	Patients	Physicians	
1. Involvement in decisions and respect for preferences	Personalized care regarding patient values • Deliberation • Phone number of the oncology nurse for support Doctor–patient relationship Time to accept the diagnosis	Personalized care regarding patient values • Deliberation Doctor–patient relationship Time to accept the diagnosis	Patients and physicians: Physicians listen to the patient, take the patient seriously, and incorporate the patient’s wishes into the treatment plan. In addition, patients should be enabled through enough time and attention given to the patient. Patients: Patients need a doctor who takes care and is available most of the time. Integrity and mutual respect are necessary for a good doctor–patient relation. Physicians: Physicians should be available to answer questions. Integrity and mutual respect are necessary for a good doctor–patient relationship. Patients and physicians: After hearing the diagnosis, most patients need time to accept the diagnosis and deal with the consequences.
2. Coordination and integration of care	Clarity about healthcare process • Follow-up • Way of informing ^1^ Multidisciplinary team Patient support groups • Fellow patients	Clarity about healthcare process • Follow-up • Way of informing ^1^ Multidisciplinary team	Patients and physicians: The healthcare path should make clear what to expect. This means all steps in follow-up should be made clear. Patients want to receive information about the healthcare process on paper or digitally. Patients and physicians: Patients need to know a person from the multidisciplinary team who can answer questions and address health-related issues. The contact-person should be regularly available. A tumor board, where physicians can discuss difficult cases with other healthcare providers, is important. Patients: When confronted with illness, patients seek professional help and advice from their doctors, and also rely on support from family members, peers, and fellow patients.
3. Clear information and communication	Information about diagnosis • Thyroid • Thyroid cancer • (Lymph node) metastases • Tumor location • Tumor size • Tumor growth • Thyroid cancer type • Clear, neutral, and stepwise way • Diagnosis being told by physician Information about prognosis • Nothing about prognosis • Survival • Treatment opportunity • No difference in treatment outcome Information about treatment options • Thyroid lobectomy • Total thyroidectomy • Radioactive iodine • Active surveillance • SDM with physician • Format of explanation ^2^ Information about risks and complications • Surgery - General - Anesthesia - Second surgery • Complications - Parathyroid gland - Vocal cord - Infection • Risk of wait and see • Psychological pressure after decision • Consequence of surgery • Recurrence Information about medication • Thyroid hormone substitution • Additional supplements • Pregnancy • Need for thyroid hormone substitution treatment • Medication adjustment • Side effects • Quality of life Information about recovery after treatment Information about recovery after treatment • Possible (negative) effects associated with each treatment - Changes in daily life - Weight loss/gain - Recurrence - Psychological aspects - Low calcium level - Fluctuations in thyroid function • Duration of recovery	Information about diagnosis • Thyroid cancer • Thyroid cancer type • Clear, neutral and stepwise way • Diagnosis being told by physician Information about prognosis • Survival • Treatment opportunity • No difference in treatment outcome Information about treatment options • Radioactive iodine • Active surveillance • SDM with physician Information about risks and complications • Surgery - Second surgery • Complications - Parathyroid gland - Vocal cord - Tracheostoma • Psychological pressure after decision • Radioactive iodine Information about medication • Medication adjustment • Quality of life Information about recovery after treatment • Duration of recovery	Patients: Patients need clear, honest, and complete information about the diagnosis. Physicians need to tell every aspect of the diagnosis. Information on the internet should be of good quality. Physicians: Patients need short and general information about the diagnosis. If not, there are concerns about the amount of information that will be forgotten. Patients and physicians: Information about the prognosis needs to be honest. To talk about treatment opportunities and outcomes is important. Especially when there is no difference in treatment outcomes. Patients: Clear and detailed information about different treatment options is important in SDM. With clear information, patients can deliberate which option fits them best. Physicians: To talk about all treatment options is important in SDM. With clear but short information, patients are able to deliberate which option fits them best. Patients and physicians: Information about the risks and complications during and after treatment is important in order to make a considered decision. The amount and consequences of complications in daily life are essential in SDM. Patients: Before patients can make a decision, it is important to have clear and extensive information about the medication. What are risks and benefits of this medication. In addition, the impact on quality of life is an important part of information about medication. Physicians: Before patients can make a decision, it is important to have complete information about the medication. Especially about the difficulties of medication adjustment. In addition, the impact of quality of life is an important part of information about medication. Patients: The possible effects after treatment are important. Patients specifically want to know what changes will take place in daily life. Physicians: Physicians mention that information about recovery is most important to patients.
4. Emotional support	Personalized psychological support for emotional problems • Reassurance • Multidisciplinary consultation		Patients: To offer psychological care to every patient is important. There should be the option to involve family and friends, for example the option to bring relatives to hospital appointments. Reassure patients through clear communication and the possibility of discussing with other healthcare providers.
5. Values	Regarding functioning in daily life • Work • Sport • Holiday • Future • Family • Quality of life • Pregnancy Regarding behavior • Self-determination • Medical decision by physician • Fear - Side-effects • Attitude • Had cancer before Medical values • Information • Decision • enetics • Medication • Quick treatment • Recovery • Conservative surgery • Extensive surgery	Regarding functioning in daily life • Quality of life Regarding behavior • Fear - Recurrence - Voice • Had cancer before Medical values • Information • Medication • Quick treatment • Conservative surgery	Patients and physicians: Values are about “what matters to me”. Values are an important part of health care decisions. Strengthening and clarifying patients’ values and preferences in the consultation is important. Values deliberation is a core step in the consultation, where the values of physicians and patients come together to reach a treatment decision

SDM—shared decision making; ^1^ To inform patients by paper or digitally/e-mail; ^2^ Treatment options have to be told in a detailed way.

**Table 3:** Quotes of patients and physicians corresponding to some of the themes for the surgery decision regarding “thyroid lobectomy or total thyroidectomy”.

Domain	Theme	Quotes Patients	Quotes Physicians
1. Involvement in decisions and respect for preferences	Time for processing the diagnosis	“It is important that you have time for processing the diagnosis and that you can think about the treatment options.”	“The diagnosis of thyroid cancer can cause anxiety and uncertainty about the future. Therefore, it is important to give patients time to process the diagnosis and make the right decision.”
2. Coordination and integration of care	Multidisciplinary team	“An important part of the decision making was the involvement of a tumor board.”“An oncology nurse is important for practical issues.”	“When we talk about a patient in our tumor board, it is possible to discuss the different treatment options and to decide if a patient is suitable for shared decision making.”“We have an oncology nurse who takes excellent care of our patients. Patients feel reassured by having a contact person.”
3. Clear information and communication	Information about prognosisInformation about medication	“I only remembered that it was treatable.”“It took a year to adjust on thyroid medication, it is necessary to receive information about this process in advance.”	“I think it is important to tell patients about their type of cancer and that there are excellent treatment options with a very good prognosis.”“It is important to say something about the adjustment of thyroid medication and the possibility of reducing quality of life.”
4. Emotional support	Personalized psychological support for emotional problems	“I wavered for a very long time, I was afraid to make the wrong treatment decision. When I discussed this with my physician, she reassured me and helped me with my decision.”	-

**Table 4:** Qualitative results for the advanced disease decision regarding “wait or start with TKIs”. Expressed information on the needs, preferences, and values of patients with and of physicians treating patients with DTC.

Information Needs for TKI Decision Making			
Domain	Theme		Description of Corresponding Items
	Patients	Physicians	
1. Involvement in decisions and respect for preferences	Personalized care regarding patient values • Deliberation • Phone number of the oncology nurse for support Doctor–patient relationship Time to accept the diagnosis	Personalized care regarding patient values • Deliberation • Phone number of the oncology nurse for support Doctor–patient relationship	Patients and physicians: Healthcare providers listen to the patient, take the patient seriously, and incorporate the patient’s wishes into the treatment plan. In addition, patients should be enabled so that there is enough time and attention for the patient. Patients: Patients need a doctor who takes care and is available most of the time. Integrity and mutual respect are necessary for a good doctor–patient relationship. Physicians: Physicians should be available to answer questions from patients. Integrity and mutual respect are necessary for a good doctor–patient relationship. Patients: After hearing the diagnosis, most patients need time to accept the diagnosis and deal with the consequences.
2. Coordination and integration of care	Clarity about healthcare process • Follow-up • Way of informing^1^ Multidisciplinary team Patient support groups • Fellow patients	Clarity about healthcare process • Follow-up • Way of informing ^1^ Multidisciplinary team	Patients and physicians: The healthcare path should make clear what to expect. This means all steps in follow-up should be made clear. Patients want to receive information about the healthcare process on paper or digitally. Patients and physicians: Patients need to know a person from the multidisciplinary team to answer questions and address health-related issues. The contact-person should be regularly available. In addition, involving oncology nurses might result in saving time during the consult with the doctor, and their involvement is described as a more personal contact. A tumor board, where physicians can discuss difficult cases with other healthcare providers. Patients: When confronted with illness, patients seek professional help and advice from their doctors, and also rely on support from family members, peers and fellow patients.
3. Clear information and communication	Information about diagnosis • Thyroid cancer • (Lymph node) metastases • Tumor growth Information about prognosis • Palliation Information about treatment options • Active surveillance • SDM with physician • TKIs • Information by physician Information about medication • Side effects • Quality of life • Dosage • Information by physician • When to provide information • When to start with medication • TKIs • Sources of information ^3^ Information about recovery after treatment • Possible (negative) effects associated with each treatment - Changes in daily life	Information about diagnosis • Tumor growth Information about prognosis • Beneft of treatment • Palliation • Communication of risk information ^2^ Information about treatment options • Active surveillance • SDM with physician • Possibility to stop with TKIs • There is no wrong decision Information about medication • Side effects • Quality of life • Dosage • Information by physician • When to provide information • When to start with medication • TKIs • Sources of information ^3^	Patients: Patients need clear, honest, and complete information about the diagnosis. Physicians need to discuss every aspect of the diagnosis. Information on the internet should be of a good quality. Physicians: Patients need short and general information about the diagnosis. If such information is not given, physicians raise concerns about the amount of information that will be forgotten. Patients: Information about the prognosis needs to be honest. After all, it is a palliative treatment. Talking about treatment opportunities and outcomes is important. Physicians: Information about the prognosis needs to be honest. Physicians want to focus on the benefits of treatment, but after all, it is a palliatve treatment. To talk about treatment opprtunities and outcomes is important. Patients: Clear and detailed information about different treatment options is important in SDM. With clear information patients can deliberate which option fits best. Physicians: To talk about all treatment options is important in SDM. With clear, but short information, patients are able to deliberate which option fits best. Patients: Before patients are able to decide, it is important to have clear and extensive information about the medication. What are the risks and benefits of this medication. In addition, the impact on quality of life is an important part of the information about medication. Physicians: Before patients are able to decide, it is important to have complete information about the medication. Especially about the side effects. In addition, the impact on quality of life is an important part of information about medication. Patients: The possible effects after treatment are important. Patients especially want to know what changes will occur in daily life.
4. Emotional support, empathy and respect	Personalized psychological support for emotional problems • Reassurance • Multidisciplinary consultation • Family	Personalized psychological support for emotional problems • Reassurance	Patients: To offer psychological care to every patient is important. There should be the option to involve family and friends, for example the option to bring relatives to hospital appointments. Reassure patients by clear communication and the possibility of discussing with other healthcare providers. Physicians: Reassure patients by clear communication and the possibility of discussing with other healthcare providers.
5. Values	Regarding functioning in daily life • Work • Sport • Holiday • Future • Family • Quality of life • Autonomy Regarding behavior • Self-determination • Deferral of medical decision to physician • Fear - Side-effects - Symptoms - Dying • Done everything Medical values • Information • Decision	Regarding functioning in daily life • Work • Holiday • Family Regarding behavior • Fear - Dying • Done everything	Patients and physicians: Values are about “what matters to me”. Values are an important part of health care decisions. Strengthening and clarifying patients’ values and preferences in the consultation is important. Values deliberation is a core step in the consultation, where values of physicians and patients come together to reach a treatment decision.

SDM—shared decision making; ^1^ To inform patients by paper or digitally/e-mail; ^2^ Visually inform patients about survival and recurrence; ^3^ Different sources of information were the pharmacy, package leaflet of the medicine, and the internet.

**Table 5:** Quotes of patients and physicians corresponding to some of the themes for the advanced disease decision regarding “wait or start with TKIs”.

Domain	Theme	Quotes Patients	Quotes Physicians
1. Involvement in decisions and respect for preferences	Time for processing the diagnosis	“The fact that you switch from an endocrinologist to an oncologist with a waiting room full of patients with cancer was a real change for me.”	
2. Coordination and integration of care	Multidisciplinary team	“A case manager can help, advise, and reassure when there are problems.”	“We discuss all patients in our tumor board and decide if whether they are a candidate for treatment with TKIs.”
3. Clear information and communication	Information about prognosis Information about medication	“You know that it is not a curative treatment and that there will be a moment when the medication is not longer working or that you have to stop because of the side effects.” “Information about the side effects was clear and detailed.” “What are the side effects and what can you expect?”	“It is important to inform patients about the possible benefit, but also that it is not a curative treatment.” “I especially talk about the side effects and quality of life.”
4. Emotional support, empathy and respect	Personalized psychological support for emotional problems	“Family means everything, my granddaughter is always aware that I cannot do everything. They give so much love.”	“Patients fear death, so your role as a physician who supports and reassures is important.”
5. Values	Regarding functioning in daily life	“Quality of life is the most important value in many different aspects of life. Maintaining quality of life is important for being able to participate in sport and work, but especially for experiencing family events.”	“What I think is important, some patients are still working and that is also quality of life. Patients just want to keep doing social things, work, to go on a holiday.”

## Data Availability

The data presented in this study are available on request from the corresponding author. The data are not publicly available due to privacy reasons.

## Appendix A

**Table A1:** Interview guide.

		**Initial Questions**	
	**Topic**	**Patients**	**Physicians**
Introduction		Explaining the interview	Explaining the interview
Information	Diagnosis Surgery TKIs	What is your diagnosis and what kind of treatment did you receive? What did the physician tell you about the diagnosis? What did the physician tell you about the surgery? OR What did the physician tell you about the TKIs?	- What do you tell patients about the diagnosis? What do you tell patients about the surgery? OR What do you tell patients about TKIs?
Evaluation	Importance Sufficient	What information provided by the physician was most the important to make a treatment decision? What aspects of the information provision did you like or dislike?	What information do you think is most important for patients? To what extent are patients informed after visiting the physician?
Experience	Decision-making capability	How should this information be presented? Would you like to be involved in decision making?	How should this information be presented? How do you feel about involving patients in decision-making during the consultation?
Values		What are important values?	What are important values for patients?
Conclusion		Do you have any additional remarks?	Are there any additional remarks?

## Appendix B

**Table A2:** Picker dimensions.

**Picker Dimensions**
1. Involvement in decisions and respect for preferences
2. Effective treatment delivered by trusted professionals
3. Continuity of care and smooth transitions
4. Involvement and support for family and carers
5. Clear information, communication and support for self-care
6. Fast access to reliable healthcare advice
7. Emotional support, empathy, and respect
8. Attention to physical and environmental needs
